# Supplementary material for: Metagenomic Changes of Gut Microbiota following Treatment of Helicobacter pylori Infection with a Simplified Low-Dose Quadruple Therapy with Bismuth or Lactobacillus reuteri
Source: Nutrients. 2022 Jul 6;14(14):2789. doi: 10.3390/nu14142789 (PMC9316840; doi:10.3390/nu14142789)

## SUPPLEMENTARY MATERIALS

**Supplementary Dataset 1.** List of identified ASVs, phyla, families, genera.

**Supplementary Table S1.** List of differential abundant phyla when comparing T0 vs. T1 and T2. Phyla with statistically significance (FDR < 0.05) and identified in at least 50% of one of the compared group are reported. In bold: taxa with significant variation in both groups.

| phylum | group A | | | | group B | | | |
|---|---|---|---|---|---|---|---|---|
| | T1 < T0 | | T2 < T0 | | T1 < T0 | | T2 < T0 | |
| | logFC (T1/T0) | FDR | logFC (T2/T0) | FDR | logFC (T1/T0) | FDR | logFC (T2/T0) | FDR |
| Bacteroidota | | | | | -8.11 | 0.00770 | | |
| **Desulfobacterota** | -9.78 | 0.00265 | | | -10.16 | 0.00547 | | |
| **Verrucomicrobiota** | -8.93 | 0.00000 | | | -8.26 | 0.02653 | | |
| **phylum** | **T1 > T0** | | **T2 > T0** | | **T1 > T0** | | **T2 > T0** | |
| | **logFC (T1/T0)** | **FDR** | **logFC (T2/T0)** | **FDR** | **logFC (T1/T0)** | **FDR** | **logFC (T2/T0)** | **FDR** |
| **Proteobacteria** | 5.16 | 0.00674 | | | 3.46 | 0.00535 | | |

**Supplementary Table S2.** List of differential abundant families when comparing T0 vs. T1 and T2. Families with statistically significance (FDR < 0.05) and identified in at least 50% of one of the compared group are reported. In bold: taxa with significant variation in both groups.

| family | group A | | | | group B | | | |
|---|---|---|---|---|---|---|---|---|
| | T1 < T0 | | T2 < T0 | | T1 < T0 | | T2 < T0 | |
| | logFC (T1/T0) | FDR | logFC (T2/T0) | FDR | logFC (T1/T0) | FDR | logFC (T2/T0) | FDR |
| Akkermansiaceae | -9.50 | 0.01010 | | | | | | |
| Anaerovoracaceae | -8.58 | 0.01010 | | | | | | |
| Barnesiellaceae | -12.09 | 0.00146 | | | | | | |
| Christensenellaceae | -11.19 | 0.02169 | | | | | | |
| **Coriobacteriaceae** | -7.29 | 0.03029 | | | -9.60 | 0.01328 | | |
| Desulfovibrionaceae | -10.57 | 0.01738 | | | | | | |
| **Eggerthellaceae** | -6.66 | 0.04200 | | | -7.49 | 0.03871 | | |
| **Marinifilaceae** | -10.46 | 0.01140 | | | -8.49 | 0.04231 | | |
| **Monoglobaceae** | -10.38 | 0.00146 | | | -7.73 | 0.01328 | | |
| Oscillospiraceae | -8.01 | 0.01293 | | | | | | |
| **Peptostreptococcaceae** | -6.76 | 0.04201 | | | -8.01 | 0.01328 | | |
| Rikenellaceae | -10.47 | 0.00281 | | | | | | |
| **family** | **T1 > T0** | | **T2 > T0** | | **T1 > T0** | | **T2 > T0** | |
| | **logFC (T1/T0)** | **FDR** | **logFC (T2/T0)** | **FDR** | **logFC (T1/T0)** | **FDR** | **logFC (T2/T0)** | **FDR** |
| **Enterococcaceae** | 12.51 | 0.00015 | | | 14.56 | 0.00908 | | |
| Lactobacillaceae | | | | | 8.14 | 0.04041 | | |
| Morganellaceae | 16.01 | 0.00000 | | | | | | |

**Supplementary Figure S1.** Alpha diversity and richness between group A and group B at the three different time points. Box plots illustrating alpha diversity (Shannon's index, left) and richness (number of observed ASVs, right) in both groups at T0 (A), T1 (B), and T2 (C). No statistically significant differences between groups were found.

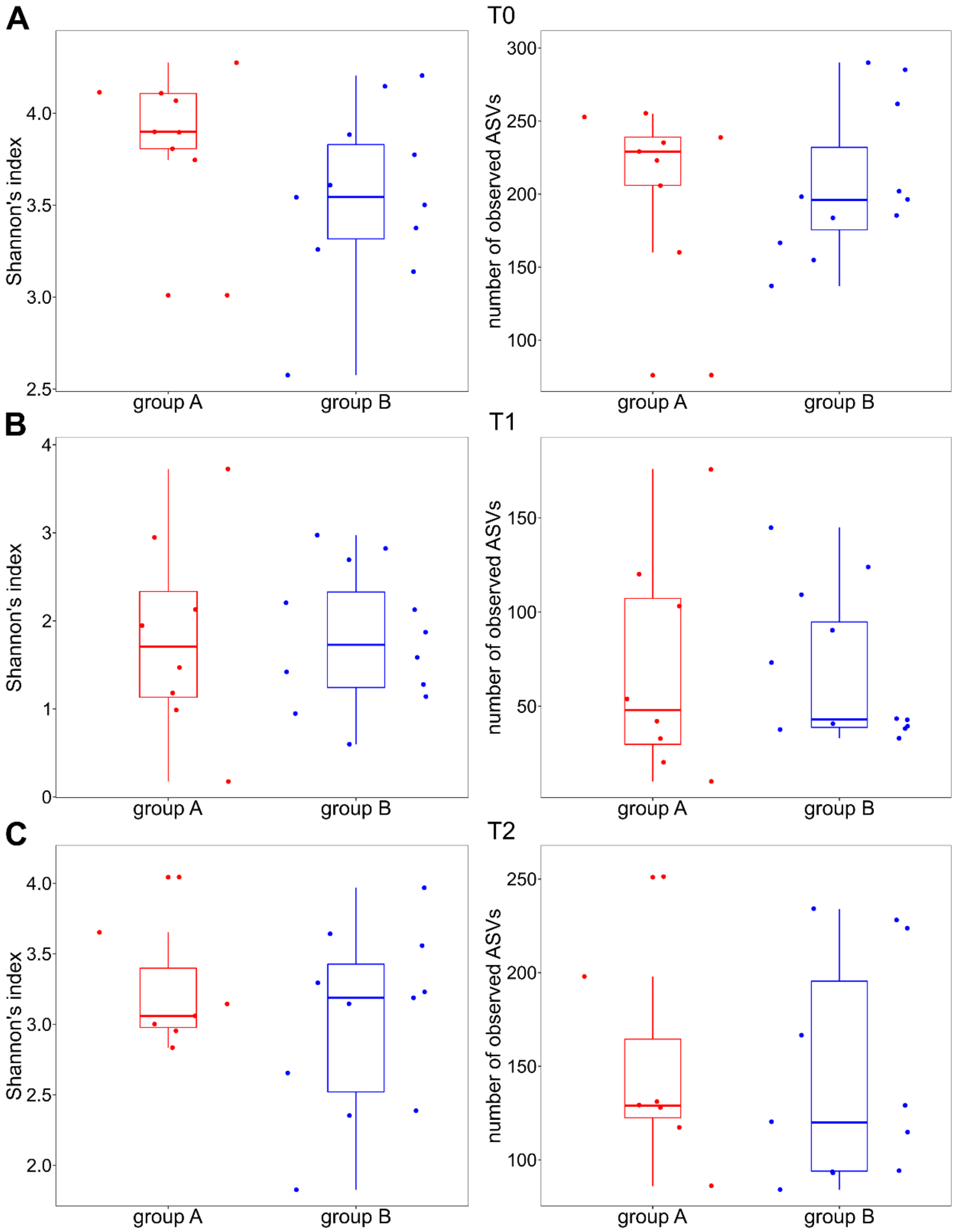

**Supplementary Figure S2.** Beta diversity between group A and group B based at the three different time points. PCoA plots showing weighted Unifrac distances based on ASV data at T0 (A), T1 (B), and T2 (C). Each dot indicates a different sample.

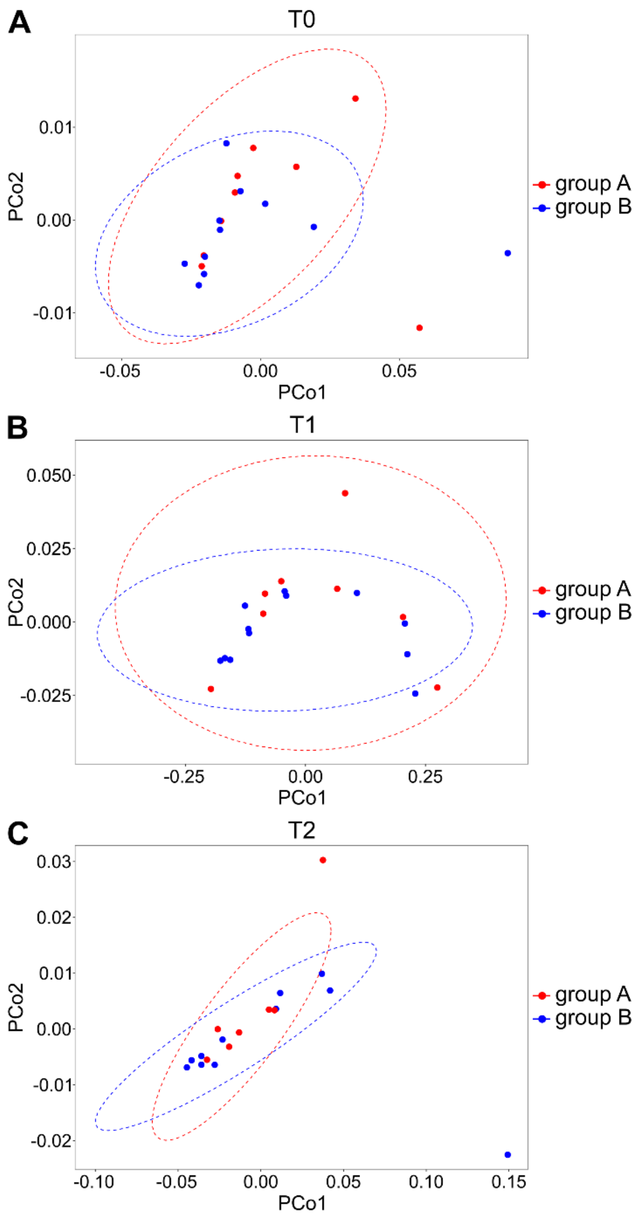

**Supplementary Figure S3.** Beta diversity between group A and group B based at the three different time points. PCoA plots showing unweighted Unifrac distances based on ASV data at T0 (A), T1 (B), and T2 (C). Each dot indicates a different sample.

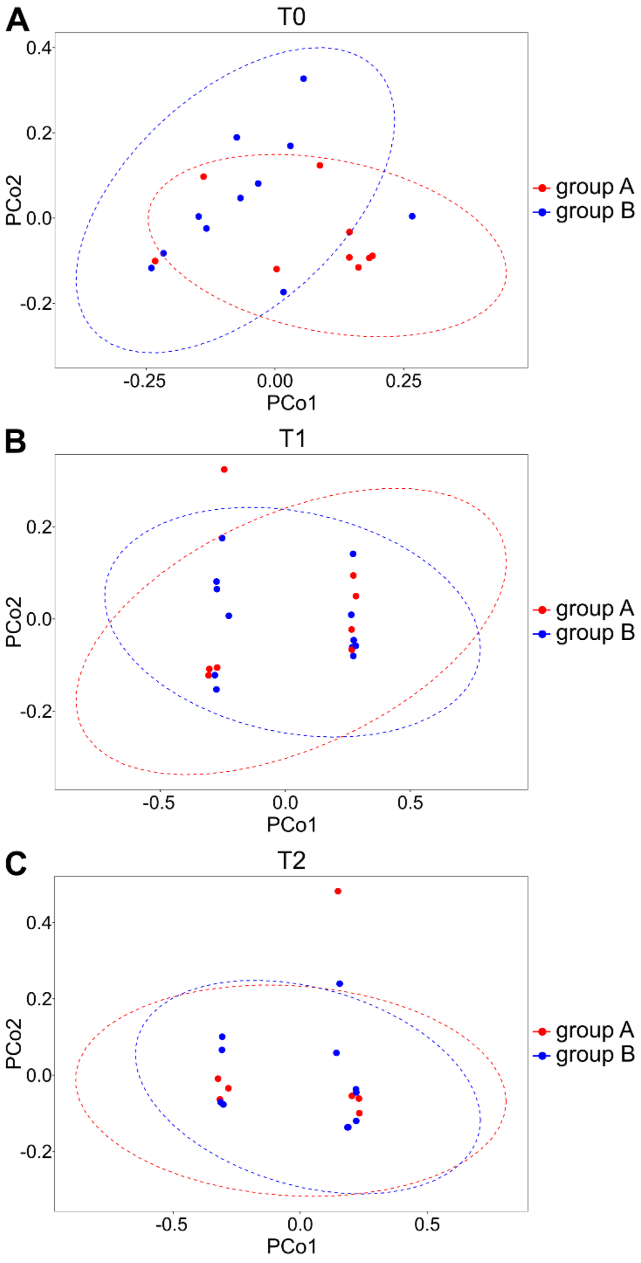

**Supplementary Figure S4.** Beta diversity within group A and group B based on the three time points. PCoA plots showing unweighted Unifrac distances based on ASV data. Each dot indicates a different sample.

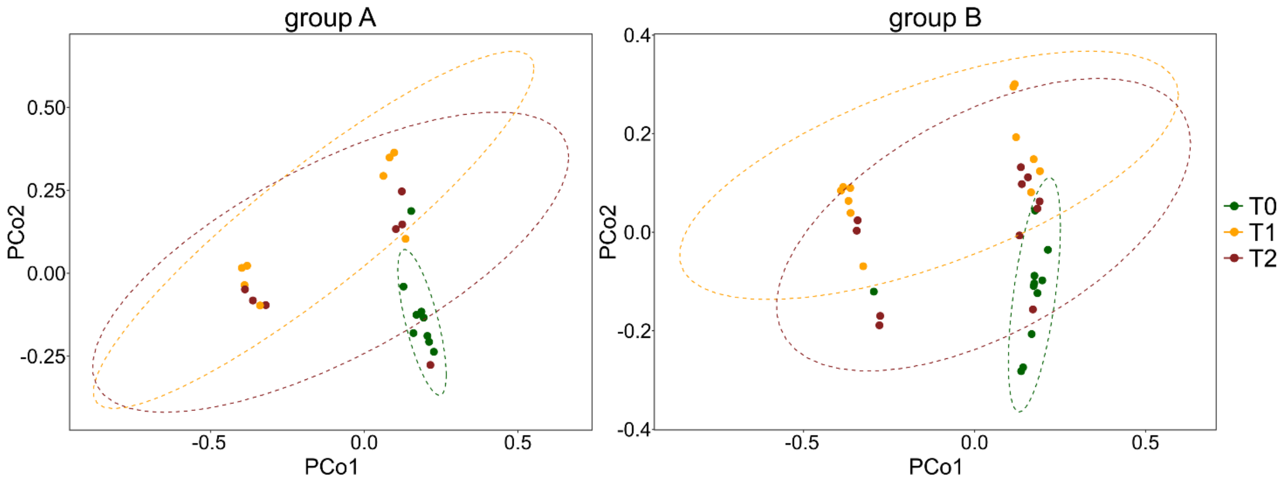

**Supplementary Figure S5.** *Lactobacillus* ASVs distribution among patients. Heatmap showing the relative abundance of the ASVs annotated as *Lactobacillus* genus identified in at least 2 samples. ASVs are ordered based on their average relative abundance Each square represents a different sample and each column represents a different patient. White squares indicate zero values, whereas barred squares indicate missing samples.

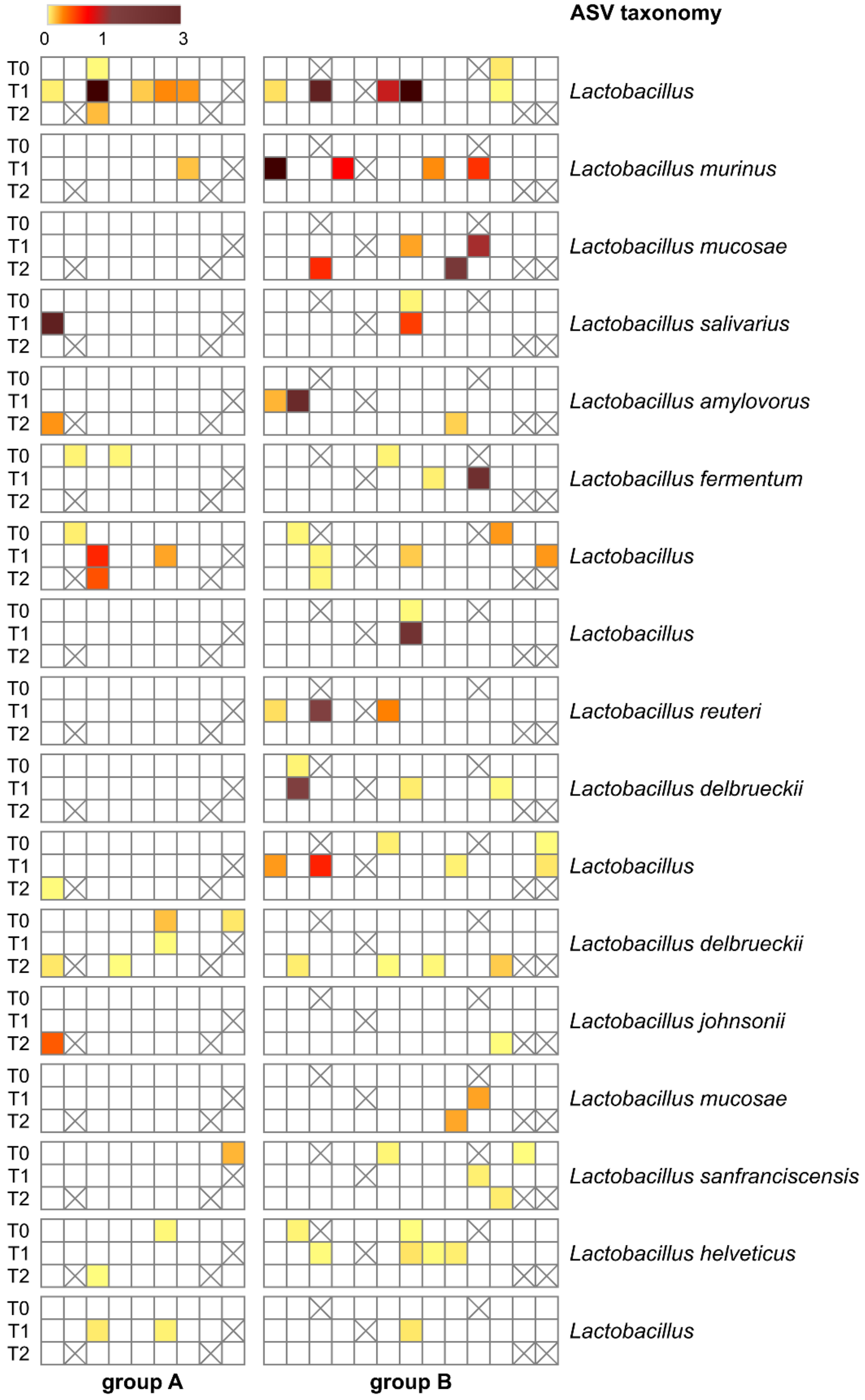

Supplement: Supplementary file 1 [file nutrients-14-02789-s001.zip › Supplementary_materials_10-05-2022.pdf]
