# Supplementary figures and images for: Metagenomic Changes of Gut Microbiota following Treatment of Helicobacter pylori Infection with a Simplified Low-Dose Quadruple Therapy with Bismuth or Lactobacillus reuteri
Source: Nutrients. 2022 Jul 6;14(14):2789. doi: 10.3390/nu14142789 (PMC9316840; doi:10.3390/nu14142789)

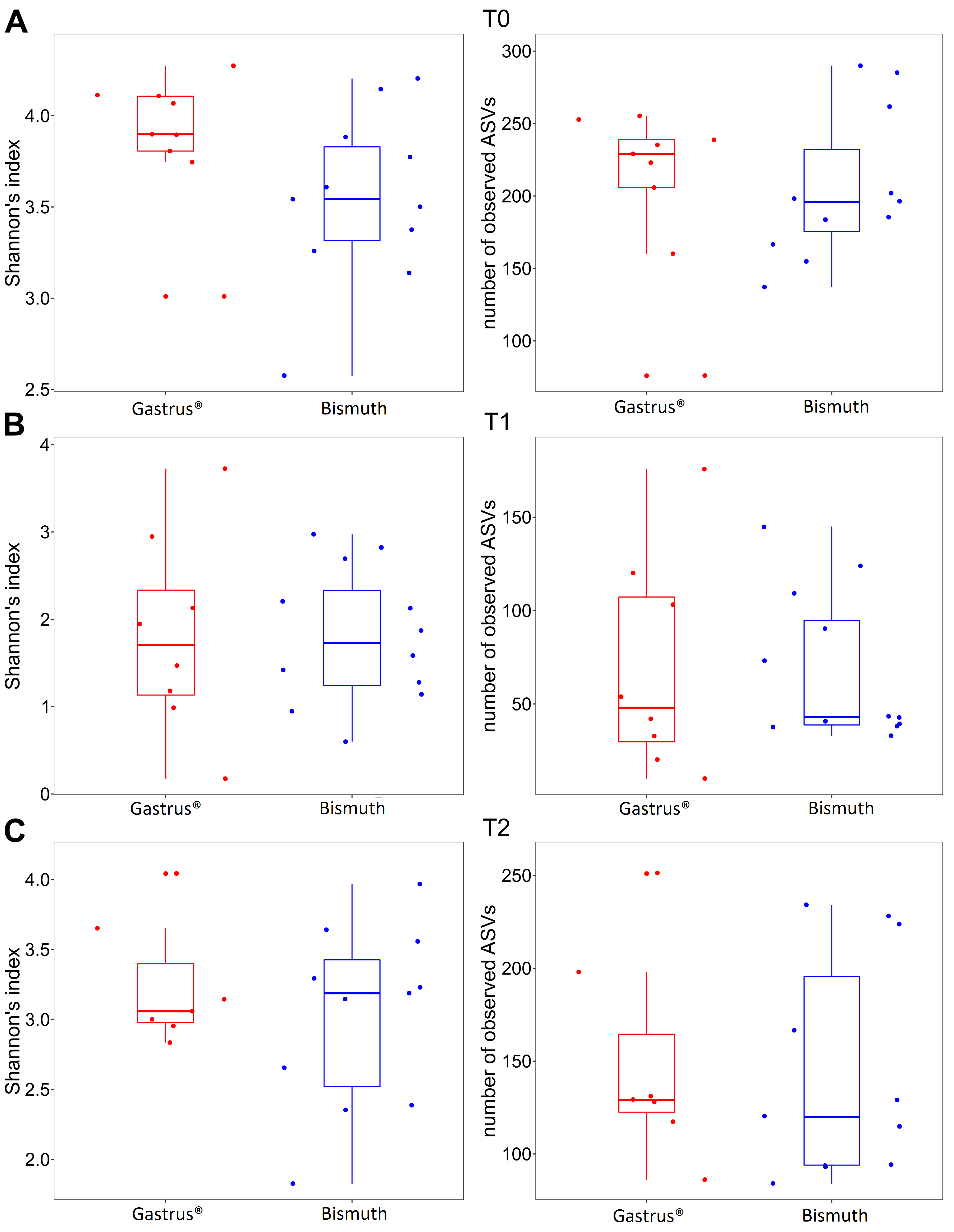

Supplement: Supplementary file 1 [file nutrients-14-02789-s001.zip › Supplementary_Figure_1.tif]

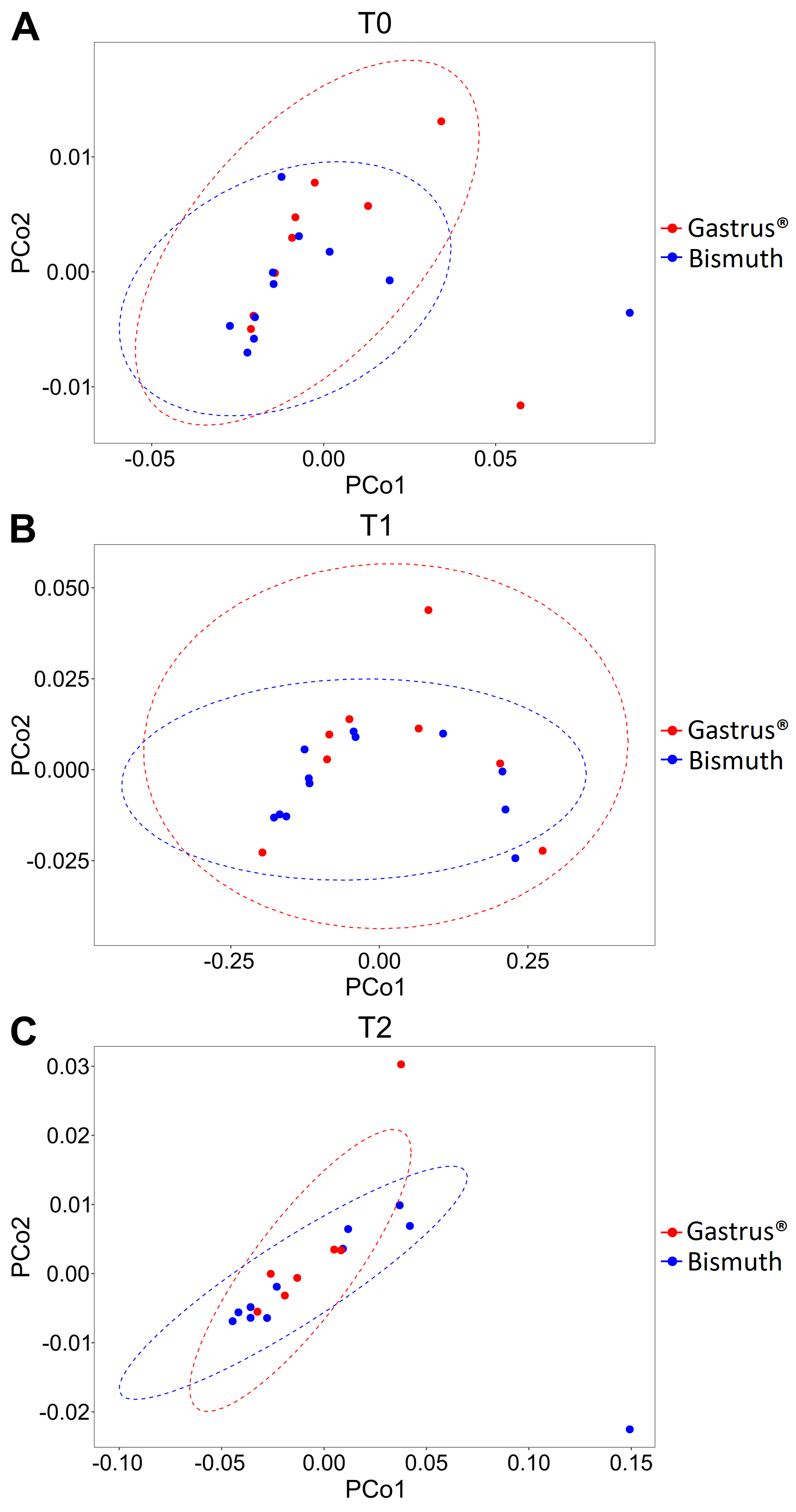

Supplement: Supplementary file 1 [file nutrients-14-02789-s001.zip › Supplementary_Figure_2.tif]

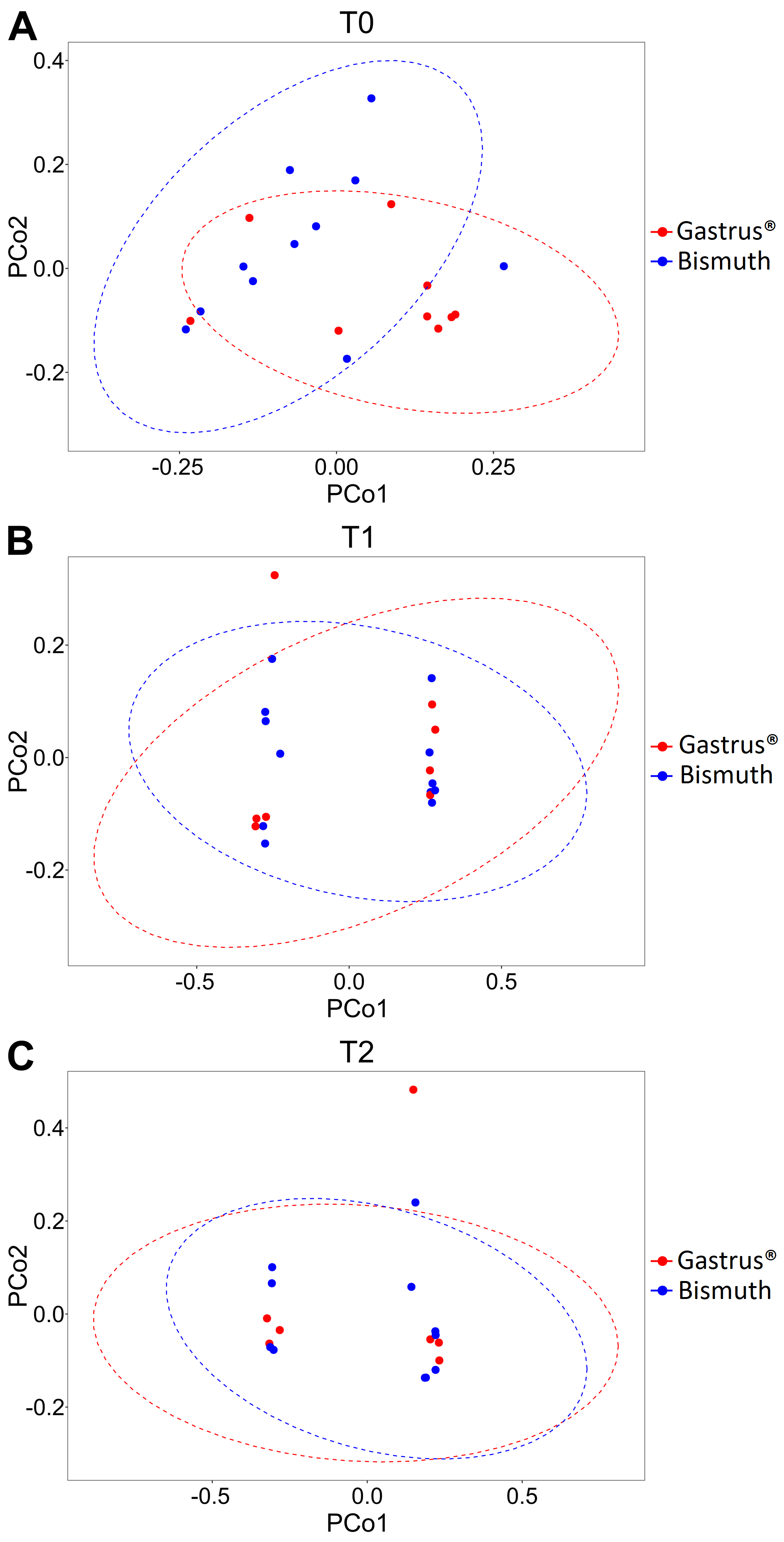

Supplement: Supplementary file 1 [file nutrients-14-02789-s001.zip › Supplementary_Figure_3.tif]

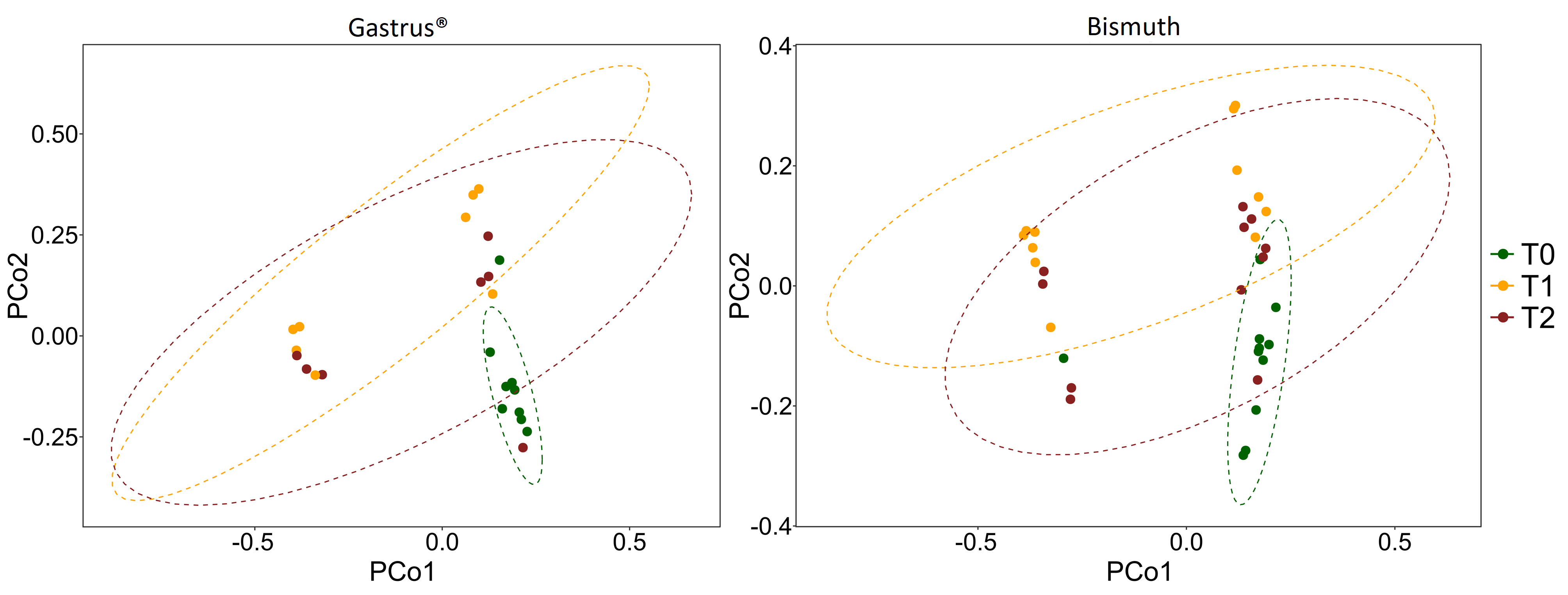

Supplement: Supplementary file 1 [file nutrients-14-02789-s001.zip › Supplementary_Figure_4.tif]

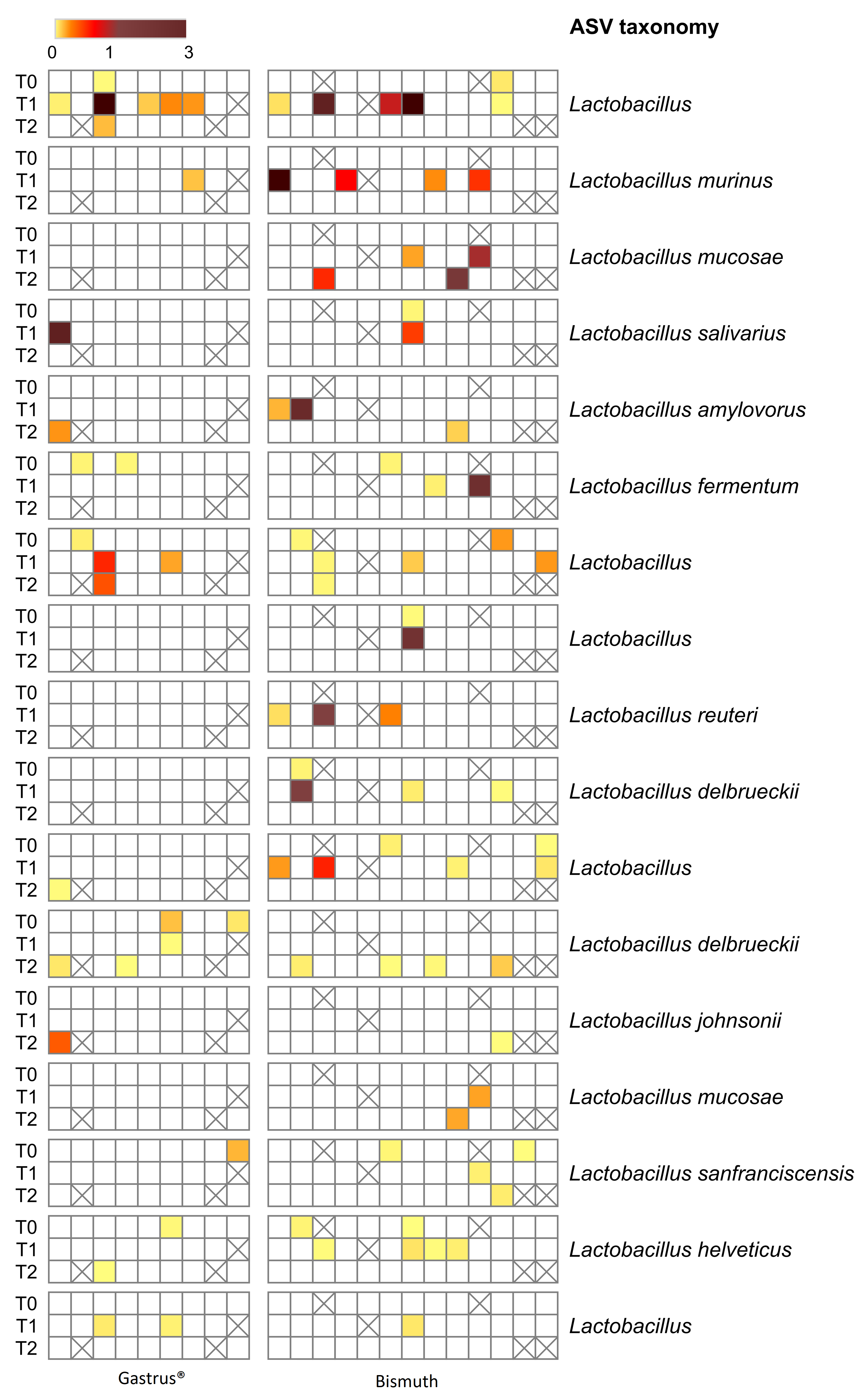

Supplement: Supplementary file 1 [file nutrients-14-02789-s001.zip › Supplementary_Figure_5.tif]
